# A Cross-Sectional Survey on COVID-19 Vaccine Hesitancy Among Parents From Shandong vs. Zhejiang

**DOI:** 10.3389/fpubh.2021.779720

**Published:** 2021-11-05

**Authors:** Yunyun Xu, Dongjuan Xu, Liyan Luo, Fengqiao Ma, Ping Wang, Hongfei Li, Qing Li, Lianyan Wei, Jiuzhou Diao, Yuanyuan Liu, Weiqiang Zhang, Xiaolei Zheng

**Affiliations:** ^1^Department of Neurology, Dongyang People's Hospital, Wenzhou Medical University, Zhejiang, China; ^2^Department of Epidemiology, The Second Hospital, Cheeloo College of Medicine, Shandong University, Jinan, China; ^3^Department of Neurology, The Second Hospital, Cheeloo College of Medicine, Shandong University, Jinan, China; ^4^Department of Dermatology, The Second Hospital, Cheeloo College of Medicine, Shandong University, Jinan, China

**Keywords:** COVID-19, parental vaccine hesitancy, PACV, Shandong, Zhejiang

## Abstract

**Introduction:** With the approval of COVID-19 vaccinations for children and adolescents in China, parental vaccine hesitancy will emerge as a new challenge with regard to the administration of these vaccines. However, little is known regarding this hesitancy as well as regional differences that may exist between parents from Shandong vs. Zhejiang.

**Methods:** To assess these issues, an online survey was conducted via a Wenjuanxing platform over the period from July 22 to August 14, 2021. Parents from Shandong and Zhejiang were recruited from Wechat groups and results from a total of 917 subjects were analyzed. Factors evaluated in this survey included socio-demographic variables, parental vaccine hesitancy, Parental Attitudes toward Childhood Vaccines (PACV) domains (behavior, safety and efficacy, general attitudes) and social support.

**Results:** Compared with those from Shandong (*N* = 443), parents from Zhejiang (*N* = 474) showed significantly higher prevalence rates of COVID-19 vaccine hesitancy (19.4 vs. 11.7%, *p* = 0.001). Multivariate logistic regression showed that yearly household incomes of ≥120,000 RMB (*p* = 0.041), medical workers (*p* = 0.022) and general attitudes of PACV (*p* = 0.004) were risk factors for vaccine hesitancy among parents from Shandong, while behavior (*p* = 0.004), safety and efficacy (*p* < 0.001) and general attitudes of PACV (*p* = 0.002) were risk factors for parents from Zhejiang. Among parents with vaccine hesitancy (*N* = 144), concerns over side effects (91.0%) and unknown effects (84.0%) of the COVID-19 vaccine were the most prevalent reasons for hesitancy. Evidence providing proof of vaccine safety (67.4%) and assurance of a low risk of being infected by COVID-19 (60.4%) were the two most effective persuasive factors.

**Conclusion:** Parents from Zhejiang showed a higher prevalence of COVID-19 vaccine hesitancy as compared with those from Shandong. Behavior, safety and efficacy, and general attitudes of PACV were the risk factors associated with this hesitancy in these parents from Zhejiang. Given the identification of the various reasons for parental vaccine hesitancy, different strategies as well as regional adjustments in these strategies will be required for an effective and convincing protocol for childhood vaccinations.

## Introduction

In July 2021, China announced that children and adolescents aged 3–17 could receive COVID-19 vaccinations (1). This announcement immediately evoked widespread attention and responses from the public, in particular parents. While vaccinations have the potential of protecting children from the COVID-19 infection and harm, some parents were hesitant for their children to be vaccinated (2, 3). In fact, this hesitancy was escalating despite findings from previous reports demonstrating that vaccinations against many major diseases have saved millions of children from death every year worldwide (4, 5). With this approval of COVID-19 vaccination for children and adolescents in China, parental vaccine hesitancy has become the focus of considerable attention and debate.

Vaccine hesitancy refers to the delay in acceptance or refusal of vaccination despite the availability of services (6). The WHO listed vaccine hesitancy as one of the top 10 global threats to health in 2019 (7). The phenomenon of vaccine hesitancy is the result of a number of complex and context specific factors, which are affected by issues such as confidence, complacency and convenience (6). According to findings from previous reports, issues of confidence refer to a lack of trust in the safety and effectiveness of the vaccine as provided by health care or government agencies (8, 9), those of complacency refer to perceptions which dismiss the value of the vaccination or necessity for vaccination (10), while those of convenience refer to difficulty in access for the vaccination. It has also been reported that social support from family, friends, colleagues and the community may be a beneficial factor for enhancing confidence (11). Importantly, understanding the reasons for parental vaccine hesitancy and developing strategies to address these issues are essential for an effective promotion of childhood and adolescent vaccinations.

As two of most populous provinces in Eastern China, Shandong and Zhejiang represent two critical regions regarding the focus of childhood and adolescent COVID-19 vaccination efforts (12). Accordingly, parents who hesitate to vaccinate their children, along with their reasons for this hesitation in these regions represent key samples of perceptions requiring attention that can likely be applicable throughout all of China, if not the world. However, only a very limited number of reports have been directed toward this issue of Chinese parental COVID-19 vaccine hesitancy for their children (13), to our knowledge, no research on this topic exists for parents from Shandong and Zhejiang provinces. Therefore, in this report an online survey on parental hesitancy from Shandong and Zhejiang provinces was conducted. With this survey, it was possible identify factors contributing to the prevalence and reasons for COVID-19 vaccine hesitancy, as well as any potential regional differences which may be present in this hesitancy.

## Materials and Methods

### Design, Subjects, and Procedure

This was a cross-sectional study performed via an online survey conducted over the period from July 22 to August 14, 2021. This study was performed a week after the Chinese government approved nationwide COVID-19 vaccinations for children and adolescents aged 3–17. With these vaccinations, public health officials suggested that the guardians of these children and adolescents provide informed consent and accompany them throughout the vaccination process (1).

From the 16 cities in Shandong Province, four cities were selected at random (Jinan, Yantai, Dongying, and Jining), and three cities were selected from the 11 cities in Zhejiang Province at random (Hangzhou, Jinhua, and Ningbo). One children and youth education center from each of these seven cities was randomly selected for this survey. To facilitate unified management, all education centers possessed their own WeChat groups, which included parents of all children. All the participants in the survey came from those WeChat groups.

Parents (≥18 years) from Shandong and Zhejiang were encouraged to participate in this online survey via a Wenjuanxing platform (https://yuyue.wjx.top/vj/OL0Dnum.aspx), which was issued on WeChat groups. This online survey contained a series of questions directed at acquiring information on socio-demographic data, clinical variables and parental willingness of vaccination against COVID-19 for their children. In order to ensure questionnaire quality, a math question (93–7 = ?) was included at the end of the survey to reduce the risk of irresponsible answers. The platform alerted participants of unanswered questions when they submitted their survey responses. This study was approved by the Medical Ethics Committee of the Dongyang Hospital affiliated to Wenzhou Medical University.

### Measurements

#### Demographic Data

Age, sex, marital status (married vs. single), education level ≤9 years (junior high school and lower) vs. > 9 years (senior high school and higher), working status (employed, unemployed), occupational classification (medical vs. non-medical workers), number of hours worked per day, yearly household income (<30,000 RMB, 30,000–120,000 RMB or ≥120,000 RMB), residence (urban vs. rural), and number of children raised (1 vs. ≥2) were collected from the questions of the online survey. Participants were also asked to provide information on the following additional issues: whether your children had a history of COVID-19 infection, vaccinations in the past 3 years, and adverse effects of vaccinations, whether family members had a history of COVID-19 infection and whether you attended any lectures about COVID-19 vaccination.

#### Parental Vaccine Hesitancy

**Figure 1:**
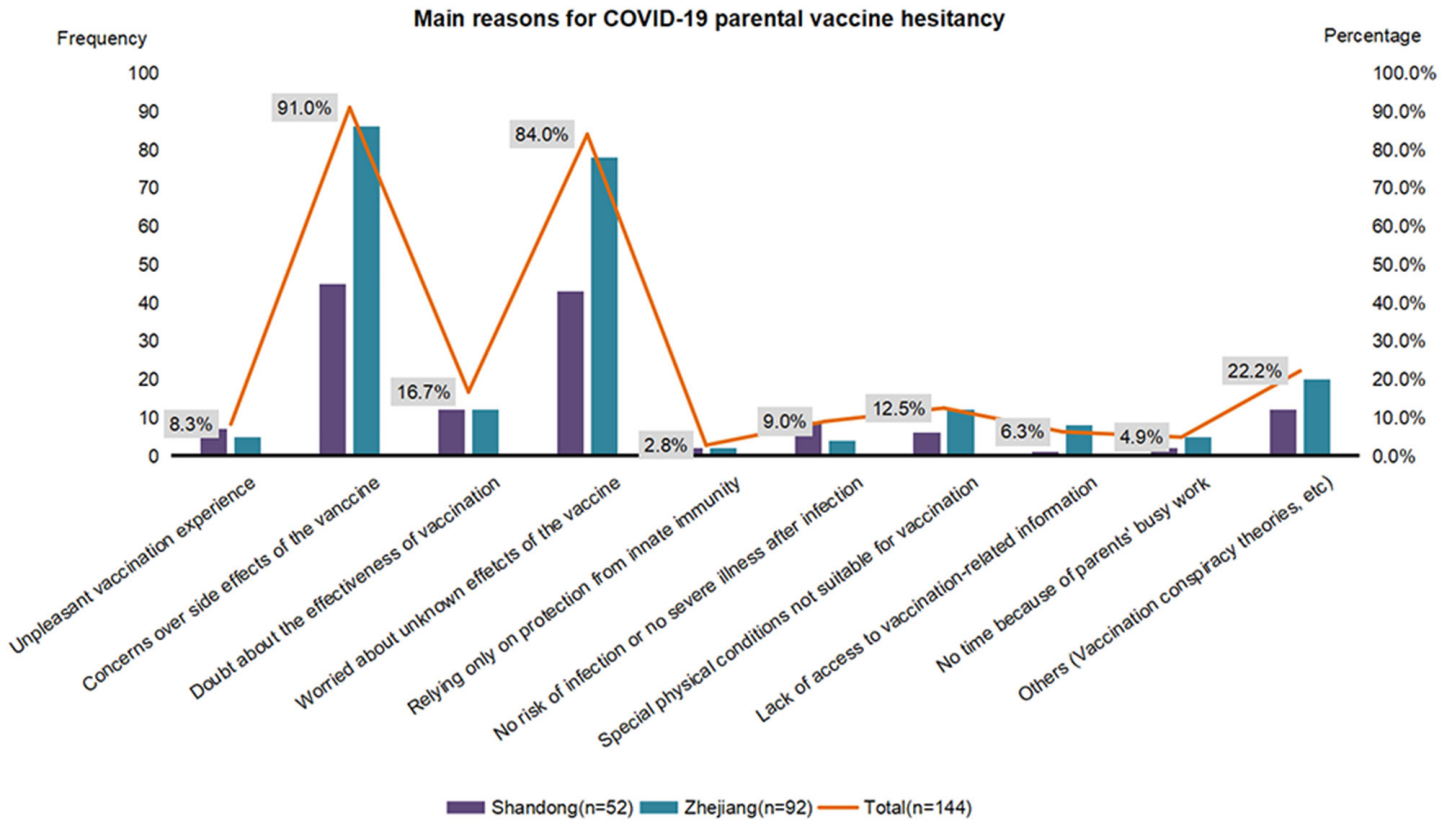
Main reasons for parental COVID-19 vaccine hesitancy in Shandong (*N* = 52) and Zhejiang (*N* = 92). COVID-19—the coronavirus disease 2019.

**Figure 2:**
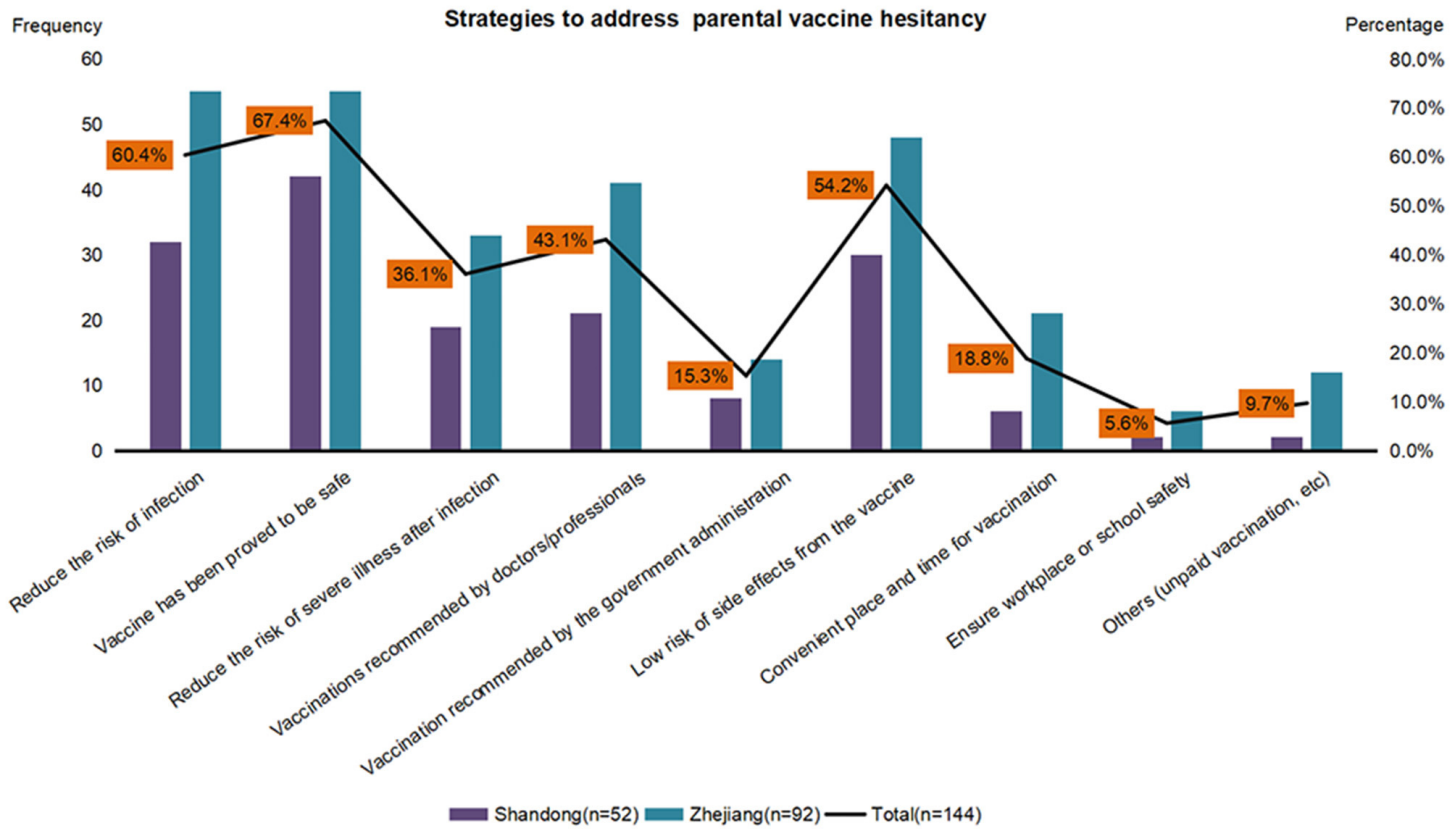
Strategies to address parental COVID-19 vaccine hesitancy in Shandong (*N* = 52) and Zhejiang (*N* = 92). COVID-19—the coronavirus disease 2019.

Our primary determination was to assess parental vaccine hesitancy, as achieved by asking participants: “If a COVID-19 vaccine was available for your children, would you like them to get it?”. Those responding “no” or “uncertain” were defined as demonstrating vaccine hesitancy. These respondents were then asked: “What are the main reasons you would not allow the vaccine for your children?”, with respondents being required to select at least one main reason as contained in a list of 10 possible answers (Figure 1). They were also asked: “Which strategies would increase the chances of you choosing the vaccination for your children?”, and were provided with a list of 9 possible answers for this question (Figure 2).

#### Parental Attitudes Toward Childhood Vaccines (PACV) Scale

The Parental Attitudes toward Childhood Vaccines (PACV) scale is a 15-item self-report tool, which can assess the behavior or attitude (doubts or concerns) associated with parental vaccine hesitancy. It is comprised of three domains, behavior (2 items; total scale scores ranging from 0 to 4), safety and efficacy (4 items; total scale scores ranging from 0 to 8) and general attitudes (9 items; total scale scores ranging from 0 to 18). Item scores were summed in an unweighted fashion to obtain a raw total score of 30. Higher scores for each domain indicate decreasing interests/perceptions regarding vaccination behavior or attitude.

#### Social Support Assessment

The Social Support Rating Scale (SSRS) is a 10-item self-report instrument to assess the level of individual social support in the last year. It was comprised of three subscales, objective support (items 2, 6, and 7), subjective support (items 1 and 3–5), and utilization of support (items 8–10). Objective support refers to direct, realistic and visible support. Subjective support refers to the perception of social support experienced whereby the individual feels the support, help and care from family, friends and colleagues. The utilization of support incarnates the level of social support. Higher scores for each subscale suggest a higher level of social support.

### Statistical Analyses

Data analyses were performed with use of the IBM SPSS Statistical Software program (version 19). All hypotheses were tested at a significance level of 0.05. χ^2^ tests or Fisher exact tests were used for categorical variables. Mann-Whitney *U*-tests were used to compare independent groups on continuous variables lacking a normal distribution. Subgroup analyses were performed for Shandong and Zhejiang parents.

Parental COVID-19 vaccine hesitancy was used as a dependent variable, while independent variables were: age, sex, marital status, working status, occupational classification, education level, number of hours worked per day, residence, yearly household income, number of children raised, history of children's COVID-19 infection, vaccination in the past 3 years, adverse effects of any vaccinations, a history of COVID-19 infection in family members, attendance at COVID-19 vaccination lectures, SSRS (i.e., objective, subjective, or utilization of support) and PACV domains (behavior, safety and efficacy, and general attitudes). There is no linear relationship between parental vaccine hesitancy and the independent variables. Multivariate logistic regression analyses were performed using stepwise variable selection with all variables entered into the model to evaluate the independent influence of COVID-19 vaccine hesitancy among Shandong and Zhejiang parents.

## Results

A total of 923 parents were contacted to participate in the online survey. Of these, results from 917 were analyzed, with subjects who failed to correctly answer the math question (6.50‰) being excluded.

Table 1 contains the socio-demographic characteristics of the respondents from Shandong (*N* = 443) vs. Zhejiang (*N* = 474) province. Parents from Zhejiang showed higher prevalence rates of COVID-19 vaccine hesitancy compared with those from Shandong (19.4 vs. 11.7%, *p* = 0.001). Parents from Shandong had higher rates of >9 years of education (*p* < 0.001), while those from Zhejiang had longer daily working hours (*p* < 0.001). Respondents from Zhejiang were more likely to live in rural areas (*p* = 0.024) and have more than one child (*p* < 0.001). Parents from Zhejiang had higher scores for the categories of safety and efficacy (*p* < 0.001), general attitudes (*p* < 0.001), subjective support (*p* < 0.001) as well as higher total PACV scores (*p* < 0.001) than those from Shandong. Statistically significant differences between Shandong and Zhejiang were also observed with regard to children's vaccination history in the past 3 years (*p* = 0.001), past adverse effects of children's vaccinations (*p* = 0.017), and behaviors of PACV (*p* < 0.001).

**Table 1:** Socio-demographic characteristics of parents from Shandong vs. Zhejiang province.

**Characteristics**	**Total**	**Shandong**	**Zhejiang**	***p***
	**(*n* = 917)**	**(*n* = 443)**	**(*n* = 474)**	
COVID-19 vaccine hesitancy	15.7 (144)	11.7 (52)	19.4 (92)	0.001
Age, years	36.98 ± 5.85	37.28 ± 4.19	36.69 ± 7.05	0.263
Male, sex	32.5 (298)	34.1 (151)	31.0 (147)	0.321
Married	93.2 (855)	92.6 (410)	93.9 (445)	0.422
Educational level >9 years	52.1 (478)	62.1 (275)	42.8 (203)	<0.001
Employed	85.6 (785)	86.9 (385)	84.4 (400)	0.277
Medical workers	6.2 (57)	6.1 (27)	6.3 (30)	0.883
Time worked per day, h	8.72 ± 2.47	8.45 ± 1.97	8.97 ± 2.85	<0.001
Household income per year, RMB				0.415
<30,000	26.8 (246)	25.1 (111)	28.5 (135)	
≥30,000 and <120,000	53.2 (488)	58.9 (261)	47.9 (227)	
≥120,000	20.0 (183)	16.0 (71)	23.6 (112)	
Living areas				0.024
Urban	40.7 (373)	44.5 (197)	37.1 (176)	
Rural	59.3 (544)	55.5 (246)	62.9 (298)	
Number of children raised ≥2	71.5 (656)	63.9 (283)	78.7 (373)	<0.001
COVID-19 infection history of the child	0.3 (3)	0 (0)	0.6 (3)	0.094
COVID-19 infection history of family member	1.2 (11)	1.6 (7)	0.8 (4)	0.306
Children's vaccination history in the past 3 years	43.6 (400)	49.2 (218)	38.4 (182)	0.001
Past adverse effects of children's vaccinations	1.4 (13)	0.5 (2)	2.3 (11)	0.017
Attended lectures about COVID-19 vaccinations	30.5 (280)	32.7 (145)	28.5 (135)	0.163
PACV				
Behavior	0.39 ± 0.73	0.46 ± 0.71	0.32 ± 0.73	<0.001
Safety and efficacy	2.76 ± 2.05	2.46 ± 1.97	3.04 ± 2.09	<0.001
General attitudes	6.83 ± 1.98	6.62 ± 1.74	7.02 ± 2.16	<0.001
Total score	9.98 ± 3.28	9.55 ± 3.09	10.38 ± 3.41	<0.001
SSRS				
Objective support	7.76 ± 2.73	7.81 ± 2.52	7.71 ± 2.91	0.333
Subjective support	25.67 ± 3.79	25.30 ± 3.20	26.01 ± 4.25	<0.001
Utilization of support	7.34 ± 2.20	7.22 ± 2.28	7.45 ± 2.13	0.125
Total score	40.76 ± 6.68	40.33 ± 6.44	41.16 ± 6.88	0.081

*Values are presented as means ± SD or percents (n). χ^2^ tests or Fisher exact tests were used for group differences of categorical variables, and Mann-Whitney tests for continuous variables. COVID-19, the coronavirus disease 2019; PACV, Parental Attitudes toward Childhood Vaccines; SSRS, Social Support Rating Scale*.

Results of the multivariate logistic regression analyses (Table 2) revealed that yearly household incomes ≥120,000RMB (odds ratio [OR], 3.76, 95% confidence interval [CI], 1.05–13.42; *p* = 0.041), medical workers (OR, 3.57; 95% CI, 1.20–10.62; *p* = 0.022) and general attitudes of PACV (OR, 1.37; 95% CI, 1.10–1.71; *p* = 0.004) were risk factors for vaccine hesitancy among parents from Shandong, while behavior (OR, 1.58; 95% CI, 1.16–2.17; *p* = 0.004), safety and efficacy (OR, 1.29; 95% CI, 1.13–1.46; *p* < 0.001), and general attitudes (OR, 1.23; 95% CI, 1.08–1.41; *p* = 0.002) of PACV, were risk factors for parents from Zhejiang. Protective factors for vaccine hesitancy in parents from Shandong were female (OR, 0.43; 95% CI, 0.22–0.86; *p* = 0.016) and ≥2 children raised (OR, 0.38; 95% CI, 0.18–0.83; *p* = 0.015), while for parents from Zhejiang, two protective factors included rural residence (OR, 0.53; 95% CI, 0.31–0.93; *p* = 0.026) and ≥2 children raised (OR, 0.53; 95% CI, 0.28–0.97; *p* = 0.040).

**Table 2:** Multivariate conditional logistic regression analysis of parental vaccine hesitancy risk in Shandong and Zhejiang provinces.

**Variables**	**OR (95%CI)**	***p***
**Shandong**		
Female	0.43 (0.22, 0.86)	0.016
Number of children raised ≥2	0.38 (0.18, 0.83)	0.015
Household income per year ≥120,000 RMB	3.76 (1.05, 13.42)	0.041
Medical workers	3.57 (1.20, 10.62)	0.022
General attitudes of PACV	1.37 (1.10, 1.71)	0.004
**Zhejiang**		
Number of children raised ≥2	0.53 (0.28, 0.97)	0.040
Living in rural	0.53 (0.31, 0.93)	0.026
Behavior of PACV	1.58 (1.16, 2.17)	0.004
Safety and efficacy of PACV	1.29 (1.13, 1.46)	<0.001
General attitudes of PACV	1.23 (1.08, 1.41)	0.002
**Total**		
Employed	2.14 (1.19, 3.85)	0.011
Household income per year ≥120,000 RMB	2.98 (1.52, 5.84)	0.002
Number of children raised ≥2	0.50 (0.33, 0.76)	0.001
Attended lectures about COVID-19 vaccinations	1.67 (1.06, 2.63)	0.028
Behavior of PACV	1.27 (1.00, 1.61)	0.048
Safety and efficacy of PACV	1.21 (1.10, 1.33)	<0.001
General attitudes of PACV	1.24 (1.11, 1.37)	<0.001

*OR, odds ratio; CI, confidence interval. Multivariate logistic regression analyses using stepwise variable selection. COVID-19, the coronavirus disease 2019; PACV, Parental Attitudes toward Childhood Vaccines*.

The main reasons why parents (52 respondents from Shandong and 92 from Zhejiang) were hesitant to vaccinate their children are summarized in Figure 1. Concerns over side effects of the COVID-19 vaccine (91.0%) and unknown effects (84.0%) were the most prevalent reasons, followed by doubt regarding the effectiveness of the vaccination (16.7%). As shown in Figure 2, when asked which factors would increase the chances of their children's vaccination, 67.4% of all parents expressing a hesitancy for vaccination reported that they would have their children vaccinated if the vaccine was proven to be safe, while 54.2% would vaccinate their children if there was an assurance for a low risk of side effects from the vaccine. Reducing the risk of COVID-19 infection was another effective persuading factor for 60.4% of the parents.

## Discussion

From this inaugural study on parental COVID-19 vaccine hesitancy as conducted in Shandong and Zhejiang provinces, a number of novel findings emerge. First, parents from Zhejiang had higher prevalence rates of COVID-19 vaccine hesitancy for their children as compared with those from Shandong. Second, behavior, safety and efficacy and general attitudes of PACV were risk factors for vaccine hesitancy among parents from Zhejiang, while yearly household incomes of ≥120,000 RMB, medical workers and general attitudes of PACV were risk factors for parents from Shandong. Third, of the main reasons for this parental hesitancy, concerns over side effects and unknown effects of the COVID-19 vaccine were the most prevalent reasons. By contrast, evidence in support of the safety for the COVID-19 vaccine was the most effective persuasive factors. These findings, offer the first data which can serve as a basis for public health strategies to diminish parental vaccine hesitancy in Shandong and Zhejiang provinces and thus increase COVID-19 vaccinations within children and adolescents.

Parental vaccine hesitancy represents a daunting problem in many countries worldwide and will significantly impact the promotion of childhood and adolescent vaccinations against the COVID-19 (14, 15). Although these COVID-19 vaccines are readily available and there exists considerable evidence indicating the effectiveness, safety and extremely low risks of side effects (16, 17), some parents throughout China remain hesitant with regard to the vaccination of their children (13, 18). Although the COVID-19 vaccination rate in Chinese adults reached more than 75% in mid-September 2021, there was a recent COVID-19 outbreak in Fujian. Worryingly, unlike previous epidemics, a large number of children were reported to be infected in this Fujian epidemic (19). In this harsh current situation, only by increasing the coverage of the vaccination for adolescents and children, they can be protected from the harm of the COVID-19. Parental vaccine hesitancy is the main obstacle to COVID-19 vaccination for children, so our study has become particularly important. In our study, 19.4% of parents from Zhejiang showed a vaccine hesitancy for the COVID-19 vaccine, which was significantly greater than that of the 11.7% of parents from Shandong. These levels of hesitancy were lower than those as reported in a previous study on the COVID-19 vaccine hesitancy among parents in Wuxi (13). Our findings suggest that not only does vaccine hesitancy among parents represent a serious issue but that regional differences exist in this hesitancy, which must be taken into account with the promotion of this COVID-19 vaccine.

Our findings also present the potential risk factors associated with this COVID-19 vaccine hesitancy as expressed by these parents from Zhejiang and Shandong. Vaccine hesitancy appears to involve an attitude (doubts or concerns) as well as a behavior (6). Mounting evidence suggests that attitudes, including not valuing or perceiving a need for the vaccination were critical issues underlying vaccine hesitancy (10, 20). Results from a previous report on parental vaccine hesitancy in Shanghai indicated that PACV could serve as a good predictor of parental vaccine hesitancy, due to its capacity to describe concerns about vaccine safety and effectiveness (21). Similarly, in our study, general attitudes, safety and efficacy and behavior of PACV were found to be risk factors for vaccine hesitancy among parents from Zhejiang. Unlike previous studies on parental COVID-19 vaccine hesitancy in China, the three domains of PACV are used as independent variables to predict vaccine hesitancy for the first time in this study (18, 22). In additional, medical workers often expressed concerns about the unknown effects of newly developed vaccines from a long-term perspective, leading to their conservative attitude toward COVID-19 vaccination (23, 24). Thus, it is not difficult to understand why medical workers in Shandong are cautious about vaccinating their children against COVID-19. Obviously, various factors related to issues of confidence and complacency, will contribute to the development of parental vaccine hesitancy. Accordingly, focusing on risk factors contributing to parental vaccine hesitancy will no doubt be critical when promoting use of these vaccinations for children and adolescents.

It is clear that the reasons for parental vaccine hesitancy are complex, varying across time, region and vaccines (6). In our study, concerns over side effects of a COVID-19 vaccine and worries about unknown effects were found to be the most frequent reasons for vaccine hesitancy, separately accounting for ~90 and 85%, respectively, of parents who were hesitant to get their children vaccinated. Recent researches on the main reasons for COVID-19 vaccine hesitancy reported that most respondents were concerned about future unknown effects and side effects related to vaccines, and some even distrusted vaccines (25, 26). Doubt regarding the effectiveness of this vaccination was another common reason for parental vaccine hesitancy in our study, a factor which was also observed in studies on vaccine hesitancy in other countries (27). Moreover, our findings reported additional reasons for parental vaccine hesitancy that include: beliefs that no risk and/or severe illness will result from this infection, special physical conditions not suitable for vaccinations and time constraints due to parental work schedules. Our current findings on the reasons for parental vaccine hesitancy provide an important new foundation for developing specific strategies in promoting the implementation of this vaccine for use in children and adolescents.

The administration of this COVID-19 vaccine within children and adolescents represents a necessary and crucial undertaking for controlling the spread of the COVID-19 epidemic (28). To accomplish this goal, different strategies should be employed to address parental vaccine hesitancy (29, 30). Of these strategies, the most important will be to disseminate knowledge about the COVID-19 vaccine along with transparent information related to side effects associated with this vaccination. Such information would substantially dispel concerns regarding side effects and build confidence in the safety of the vaccine (31). Moreover, increasing public awareness would highlight the importance of this vaccination and contribute to changes in parental negative attitudes toward childhood vaccinations (32). In this regard, new media and short videos which promote these vaccinations may greatly aid in their acceptance (33). The inception of a free, full coverage COVID-19 vaccination for these children and adolescents as initiated within China in July 2021 should maximize the potential for protection against this epidemic.

## Limitations

This study has several limitations. First, the inadequacies associated with such a cross-sectional design cannot be avoided, and a survey on changes in parental vaccine hesitancy across different periods should be encouraged. Second, the sample size in the survey was relatively small which may, in part, be due to some parents voluntarily opting out of participation in this study. Finally, as it was not possible to identify the characteristics of parents outside the survey, we cannot verify that the participants of this study were representative of all the parents from Shandong or Zhejiang.

## Conclusion

In the promotion of childhood and adolescent vaccinations against COVID-19, parents from Zhejiang showed a higher prevalence of vaccine hesitancy compared with those from Shandong. Behavior, safety and efficacy, and general attitudes of PACV were risk factors for these parents from Zhejiang. As various reasons exist for parental vaccine hesitancy, multiple strategies should be employed for promoting vaccinations. In particular, strengthening knowledge regarding the safety and importance of the vaccine would greatly help in establishing confidence for this vaccine as administered within children and adolescents.

## Data Availability Statement

The raw data supporting the conclusions of this article will be made available by the authors, without undue reservation.

## Ethics Statement

The studies involving human participants were reviewed and approved by Dongyang People's Hospital, Wenzhou Medical University. The participants provided their written informed consent to participate in this study.

## Author Contributions

YX and XZ: conception and design. YX, DX, FM, PW, HL, QL, LW, JD, YL, WZ, and XZ: conduction. LL: statistical analysis. YX, DX, FM, PW, HL, QL, and XZ: administrative, technical, or material support. YX and XZ: drafting of the manuscript. XZ: critical revision of the manuscript for important intellectual content. All authors read and approved the final paper.

## Conflict of Interest

The authors declare that the research was conducted in the absence of any commercial or financial relationships that could be construed as a potential conflict of interest.

## Publisher's Note

All claims expressed in this article are solely those of the authors and do not necessarily represent those of their affiliated organizations, or those of the publisher, the editors and the reviewers. Any product that may be evaluated in this article, or claim that may be made by its manufacturer, is not guaranteed or endorsed by the publisher.
